# Adherence to Mediterranean Diet and Response to an Exercise Program to Prevent Hospitalization-Associated Disability in Older Adults: A Secondary Analysis from a Randomized Controlled Trial

**DOI:** 10.1007/s12603-023-1929-6

**Published:** 2024-01-04

**Authors:** A. Tor-Roca, J. Mayordomo-Cava, C. Andres-Lacueva, J.A. Serra-Rexach, Mireia Urpi-Sarda

**Affiliations:** 1 Biomarkers and Nutrimetabolomics Laboratory, Department of Nutrition, Food Science and Gastronomy, Food Innovation Network (XIA), Institute for Research on Nutrition and Food Safety (INSA-UB), Food and Nutrition Torribera Campus, Faculty of Pharmacy and Food Sciences, University of Barcelona, 08028, Barcelona, Spain; 2 CIBER of Frailty and Healthy Aging (CIBERFES), Instituto de Salud Carlos III, 28029, Madrid, Spain; 3 Geriatric Department, Hospital General Universitario Gregorio Marañón, 28007, Madrid, Spain; 4 Health Research Institute Gregorio Marañón (IiSGM), 28007, Madrid, Spain; 5 School of Medicine, Complutense University of Madrid, 28040, Madrid, Spain; 6 C/Prat de la Riba, 171, 08921, Santa Coloma de Gramenet, Spain

**Keywords:** Mediterranean dietary pattern, phenolic compounds, hospitalization-associated disability, functional ability, activities of daily living

## Abstract

**Objectives:**

To investigate the relationship between Mediterranean diet (MedDiet) adherence and response to an exercise and health education program to prevent hospitalization-associated disability (HAD) in acutely hospitalized older adults.

**Design:**

Randomized controlled trial.

**Setting and Participants:**

Secondary analysis of a subset of 109 participants from AGECAR-PLUS study with available data on MedDiet adherence (mean age 87, and range 75–98).

**Intervention:**

Participants were randomized into the control group (n = 46, usual care) or the intervention group (n = 63, supervised exercise and health education) at admission.

**Measurements:**

MedDiet adherence was measured with MEDAS and through urinary total polyphenols (UTP). Functional status was assessed with the Barthel Index.

**Results:**

At discharge, patients in the intervention group who had low levels of MedDiet or UTP showed an increase in functional status [adjusted mean (95% CI) = 77.8 (70.8–84.8) points, p = 0.005, and adjusted mean (95% CI) = 78.0 (68.3–87.7) points, p = 0.020, respectively].

**Conclusion:**

Older individuals over age 75 with low MedDiet adherence were likely to benefit more from a physical exercise and health education intervention.

## Introduction

**I**n older patients, acute medical illness that necessitates hospitalization may often precipitate disability even when such illness is successfully treated. This leads to the subsequent inability to live independently and complete basic activities of daily living (ADLs) (1). Hospitalization-associated disability (HAD, i.e. loss of the ability to perform one or more ADLs independently at discharge) occurs in approximately one third of patients older than 70 and is associated with an increased length of stay, higher use of resources, institutionalization, hospital readmissions, long-term disability and mortality (2). Physical exercise interventions have proven viable and safe in acutely hospitalized older patients and are effective for enhancing their functional status at discharge as well as for reducing the cost and length of hospitalization (3, 4). On the other hand, a healthy dietary pattern has been in the spotlight because of its effects on healthy aging (5). In fact, recent evidence suggests that healthy dietary patterns, such as the Mediterranean diet (MedDiet), the MIND diet, and the DASH diet, may reduce the risk of physical impairment, mobility disability, and frailty in community-dwelling and institutionalized older adults (6, 7, 8). The concentration of urinary total polyphenols (UTP) is a well-known objective biomarker of total dietary polyphenol exposure and a proxy measure of dietary fruit and vegetable intake (9). Moreover, UTP depends on the bioavailability of polyphenols, which tends to decrease during aging and may consequently impact their biological activity (10). Despite this interest, little research has been devoted to evaluating the relationship between UTP and age-related comorbidities, although this shows promising evidence. For instance, high UTP levels have been associated with a lower risk of physical performance decline (i.e. walking speed, rising from a chair, and standing balance), frailty, exhaustion, weakness, and cognitive decline in community-dwelling old adults (9, 11). However, to the best of our knowledge, no studies have explored the effect of the MedDiet and UTP on functional status in acute hospitalized older adults.

Hence, in this paper, we sought to investigate the relationship between MedDiet adherence and the response to an exercise and health education program aimed at preventing HAD in acutely hospitalized adults aged over 75 years during hospitalization.

## Materials and methods

### Study design

The Activity in GEriatric acute CARe Plus (AGECAR-PLUS) Health Education is a randomized control trial to assess the effectiveness of an intrahospital exercise and health education program during short hospital stays for improving functional capacity of patients aged 75 years or older. The details of the clinical trial (NTC03604640) are described elsewhere (https://clinicaltrials.gov/ct2/show/NCT03604640). Briefly, 260 patients (> 75 years) admitted to the acute care for older patients (ACE) unit of the Hospital General Universitario Gregorio Marañón (Madrid, Spain) were considered eligible to participate in the study and were thus randomized to a control group or intervention group. Participants allocated in the control group received standard hospital care. Participants allocated in the intervention group performed, during hospitalization, a multicomponent training program (30 minutes per session, twice a day on weekdays, lower limb strength training, balance training, walking, and inspiratory muscle training) and health education. Health education consisted of several informational activities. Each activity session taught the patient and caregiver how to perform the exercises to ensure continuity after discharge. Eligible participants were included in the control or intervention group in a time-dependent manner (i.e. using 4-week blocks) to avoid the co-presence of patients from both groups in the unit, so that participants were blinded to actual group assignment (Supplementary Figure 1).

**Figure 1:**
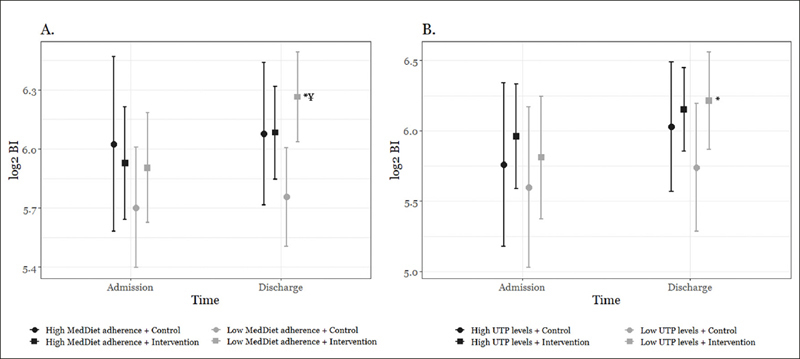
Functional Status at Admission and at Discharge of Participants in Both Control and Intervention Groups According to MedDiet Adherence (A.) and UTP Levels (mg GAEs/L) at Admission (B.) (Intention-to-Treat Analysis)

Note. BI, Barthel Index; CI = confidence interval; MedDiet = Mediterranean diet; UTP = urinary total polyphenols. Models are adjusted for sex, age, BMI at admission, place of living, independent ambulation, Charlson Comorbidity Index, polypharmacy (≥ 7), cause of hospitalization (admission diagnosis) and frailty phenotype. In the UTP levels model, we further adjusted for creatinine clearance at admission and chronic kidney disease. A low level of MedDiet adherence consisted in a MEDAS score < 9 and a high level of MedDiet adherence consisted in a MEDAS score ≥ 9 at admission. Low levels of UTP consisted in ≤ 11.1 mg GAEs/L and high levels of UTP consisted in > 11.2 mg GAEs/L at admission. Functional status was assessed with the Barthel Index of independence in ADLs, which uses a total 0 to 100 score and includes the following 10 basic ADLs: feeding, transferring, walking on level surfaces, stair climbing, bowel and bladder control, toileting, bathing, grooming and dressing (15). * Different from admission, * p < 0.05; ¥ Different from control group, ^¥^ p < 0.05 (ANOVA for repeated measures with Bonferroni post hoc test).

The Institutional Review Board approved the protocol (Hospital Universitario Gregorio Marañón, Madrid, Spain), and all participants provided written informed consent. When it was not possible to obtain the informed consent directly from a patient for medical reasons (e.g. impaired cognitive function), proxy consent was obtained from their relatives. All procedures were performed in accordance with the ethical standards from the Declaration of Helsinki and its later amendments.

In this current work, we conducted a secondary analysis with 109 older participants from AGECAR-PLUS trial who had the MEditerranean Diet Adherence Screener (MEDAS) or urine sample available at admission (63 and 46 individuals in the intervention and in the control group, respectively) (Supplementary Figure 1).

### Participants

The participant inclusion criterion was patients aged 75 and older admitted to the ACE unit. The exclusion criteria were: nonambulatory or dependent in all basic ADLs at baseline (i.e. two weeks before admission, as assessed by retrospective interview); having an unstable cardiovascular disease (or any other major medical condition contraindicating exercise), terminal illness, or severe dementia; expected length of hospitalization < three days; having a scheduled admission (which was usually associated with a length of hospitalization < three days) (4); or being transferred from another hospital unit.

### Measurement of Mediterranean diet adherence

#### Determination of total polyphenols in urine samples

Spot urine samples were collected at admission (Supplementary Figure 1). Urine samples were aliquoted, processed, and stored at −80°C until analysis. UTP were analyzed using Folin-Ciocalteu reagent after solid-phase extraction utilizing Oasis HLB 96-well plates (Waters, Milford, MA), as described elsewhere (9). Total polyphenol results were further normalized with reference to the urinary refractive index to account for interindividual differences in the hydration status and micturition frequency (10). UTP concentrations were expressed as mg gallic acid equivalents (GAEs)/L (9). UTP has previously been positively correlated with adherence to the MedDiet (12). UTP levels were further divided into two median-based groups to categorize UTP levels as low (≤ 11.1 mg GAEs/L) and high (> 11.2 mg GAEs/L).

#### MEDAS questionnaire

At admission, the validated 14-item MEDAS questionnaire was used to briefly assess adherence to a traditional MedDiet (13). This questionnaire reports the habitual frequency of consumption or amount consumed of 12 main components of the MedDiet and two food intake habits considered characteristic of the Spanish MedDiet (13). Each of the 14 items was scored 1 or 0, depending on whether or not patients met each MedDiet condition. One point was assigned for using olive oil as the principal source of fat for cooking; for preferring white meat over red meat; for consuming ≥ 4 tablespoons of olive oil/day (including that used in frying, salads, meals eaten away from home, etc.); ≥ 2 servings of vegetables/day; ≥ 3 servings of fruit (including freshly squeezed juice)/day; < 1 serving of red meat, hamburger, or sausages/day; < 1 serving of animal fat (i.e. butter, margarine, or cream)/day; < 1 cup of sugar-sweetened and/or carbonated beverage/day; ≥ 7 cups of red wine/week; ≥ 3 servings of pulses/week; ≥ 3 servings of fish or seafood/week; < 2 servings of commercial pastry/week; ≥ 3 servings of nuts/week; ≥ 2 servings/week of a dish with a traditional sauce of tomatoes, garlic, onion, or leeks sautéed in olive oil (13). MEDAS scores were divided into two groups to categorize adherence as low (scores < 9) and high (scores ≥ 9) (14).

### Endpoint assessment

The functional status with regard to the basic ADLs was determined at admission and at discharge. Functional status was evaluated with the Barthel Index (BI), which measures patients' ability to independently perform ten basic ADLs [feeding, transferring from bed to chair and back, walking on level surfaces, stair climbing, bowel and bladder control, using the toilet, bathing, grooming (e.g. tooth brushing, shaving), and dressing] (15). The sum score for this index ranges from 0 (total dependence) to 100 (complete independence) points.

Those medical and research staff who were in charge of the outcome assessment were not involved in supervising the intervention. However, assessors and care providers were not blinded to the assigned intervention.

### Other variables

Upon admission, demographic (age, sex, place of living) and clinical characteristics [diagnosis at admission, presence of other geriatric syndromes (dementia, depression, falls, chronic pain, malnutrition (having ≤ 7 of the 14 Mini Nutritional Assessment–Short Form criteria (16)), urinary incontinence, frailty phenotype (having ≥ 3 of the five Fried's criteria (17))), comorbidities (assessed by means of the Charlson Comorbidity Index (18)), independent ambulation (considered in the event of a functional ambulatory classification (FAC) = 4 (19)), lower extremity function and mobility (assessed by the Short Physical Performance Battery (SPPB) (20)), and polypharmacy (taking ≥ 7 drugs)] were also assessed.

### Statistical analysis

Baseline characteristics of the participants were expressed as means and standard deviations (SD) for variables with normal distribution, medians and interquartile ranges (IQR) for variables with asymmetric distribution, and percentages for categorical variables. UTP and BI were not normally distributed and were thus normalized using a Box–Cox transformation (α = 0.00001 and λ = 0.25) (9) and a log2 transformation, respectively.

Differences in characteristics between groups at admission were analyzed using the Student's t-test, the chi-square test, or the Mann–Whitney U test, when appropriate.

One-factor analysis of variance (ANOVA) for repeated measures with the Bonferroni post hoc test was used to compare changes in BI in response to the intervention treatment and to the control group. In addition, it was also used to compare changes in functional status considering low and high levels of the following variables: a) adherence to MedDiet at admission and b) concentrations of UTP at admission. We studied the effect of the interaction of two factors: time as a within-participants factor (admission and discharge) and groups after adjustment for sex, age, body mass index (BMI) at admission, place of living, independent ambulation, Charlson Comorbidity Index, polypharmacy (≥ 7), cause of hospitalization (admission diagnosis), frailty phenotype, and additional creatinine clearance at admission, and chronic kidney disease. To assess the effects of individual factors, we finally calculated the differences between discharge and admission values for the BI and then we applied a one-factor analysis of covariance (ANCOVA) test adjusting for the above-mentioned variables and for functional status at admission.

Intention-to-treat analysis were performed using the methods of the last observation carried forward/backward (21, 22), and sensitivity analysis were performed using a complete-case analysis. We did not impute missing data of covariates, and thus only available data were used for analysis. Between-and within-group differences were expressed as estimated means and 95% CI. Finally, it should be noted that the AGECAR-PLUS trial was powered to assess the effectiveness of an intrahospital exercise and health education program for improving functional capacity of older patients, therefore, sample size calculations were based on the main outcomes of the trial. However, in this study, we performed an exploratory analysis of data acquired from the AGECAR-PLUS trial, thus, the statistical power has been calculated (23).

All statistical analyses were performed using the SPSS software package (SPSS 25.0, IBM, NY) and the significance level was a p < 0.05.

## Results

The participants' characteristics according to intervention and MedDiet adherence groups are shown in Table 1 (intention-to-treat analysis) and in Supplementary Table 1 (complete-case analysis). The mean (SD) age of the participants was 87 (5) (range 75–98) and 46% were female. The median (IQR) length of hospitalization was 5.0 (4.0) days. No general between-group differences were found at the start of the study for sociodemographic and other clinical characteristics (all p > 0.05; Table 1 and Supplementary Table 1) although in the intervention group BMI at admission was slightly different between MedDiet adherence groups (p < 0.05). Participants in the intervention group performed a mean (SD) of 7.1 (5.8) training sessions. No adverse effects or falls were recorded during the intervention. BI [median (IQR)] at admission was similar among participants in both control and intervention groups (Table 1 and Supplementary Table 1). The mean (SD) adherence to the MedDiet was 7.9 (2.1) and it was similar between groups (Table 1). Individuals showed similar UTP levels at admission between groups (Table 1 and Supplementary Table 1).

**Table 1:** Main Characteristics of the Study Participants by Intervention Group and Mediterranean Diet Adherence (Intention-to-Treat Analysis)

**Variables**	**Control**		**Intervention**		**p**	**Control**		**Intervention**		**p**
	**Low adherence**^f^**(n=31)**	**High adherence**^f^**(n=14)**	**Low adherence**^f^**(n=34)**	**High adherence**^f^**(n=26)**		**Low UTP levels**^f^**(n=16)**	**High UTP levels**^f^**(n=14)**	**Low UTP levels**^f^**(n=19)**	**High UTP levels**^f^**(n=21)**	
Age [mean (SD)], y.	88.2 (4.8)	85.8 (4.2)	87.1 (4.3)	87.3 (4.7)	0.421	86.8 (4.7)	86.9 (5.6)	87.2 (4.6)	87.2 (4.5)	0.990
Female [n (%)]	16 (51.6)	7 (50.0)	17 (50.0)	9 (34.6)	0.565	9 (56.3)	7 (50.0)	7 (36.8)	8 (38.1)	0.601
BMI at admission^§^ [mean (SD)], kg/m^2^	20.5 (3.2)^ab^	21.2 (4.1)^ab^	22.9 (3.9)^a^	20.2 (2.4)^b^	0.014	19.8 (2.5)	23.3 (4.5)	21.6 (2.6)	20.9 (4.0)	0.088
Living at home [n (%)]	28 (90.3)	14 (100.0)	32 (94.1)	25 (96.2)	0.673	16 (100.0)	13 (92.9)	17 (89.5)	20 (95.2)	0.658
Charlson comorbidity index^§^ [mean (SD)]	2.8 (1.8)	2.4 (1.7)	3.2 (1.9)	3.7 (2.7)	0.232	2.1 (2.2)	2.7 (1.5)	3 (2.0)	3.4 (2.2)	0.287
Geriatric syndromes										
Frailty phenotype^§^ [n (%)]	15 (71.4)	8 (80.0)	14 (50.0)	16 (66.7)	0.255	8 (72.7)	9 (90.0)	10 (58.8)	14 (66.7)	0.407
Urinary incontinence^§^ [n (%)]	18 (60.0)	6 (42.9)	17 (50.0)	12 (46.2)	0.659	10 (62.5)	6 (42.9)	12 (63.2)	11 (52.4)	0.624
Depression^§^ [n (%)]	11 (36.7)	7 (50.0)	15 (44.1)	9 (34.6)	0.737	8 (50.0)	7 (50.0)	5 (26.3)	10 (47.6)	0.401
Falls^§^ [n (%)]	13 (43.3)	3 (21.4)	10 (29.4)	8 (30.8)	0.464	6 (37.5)	4 (28.6)	7 (36.8)	5 (23.8)	0.765
Chronic pain^§^; [n (%)]	10 (33.3)	2 (14.3)	9 (26.5)	10 (38.5)	0.407	6 (37.5)	3 (21.4)	9 (47.4)	6 (28.6)	0.416
Malnutrition^b§^ [n (%)]	6 (24.0)	5 (35.7)	2 (7.4)	8 (33.3)	0.074	5 (35.7)	2 (16.7)	4 (23.5)	6 (30.0)	0.301
Dementia^§^ [n (%)]	5 (16.7)	1 (7.1)	2 (5.9)	4 (15.4)	0.507	1 (6.3)	1 (7.1)	3 (15.8)	3 (14.3)	0.756
Polypharmacy (≥7)^§^ [n (%)]	20 (66.7)	12 (85.7)	28 (82.4)	21 (80.8)	0.360	12 (75)	10 (71.4)	16 (84.2)	18 (85.7)	0.693
Main admission diagnosis [n(%)]					0.834					0.338
Infectious	11 (35.5)	5 (35.7)	9 (26.5)	7 (26.9)		4 (25.0)	6 (42.9)	4(21.1)	5 (23.8)	
Circulatory	8 (25.8)	6 (42.9)	7 (20.6)	7 (26.9)		6 (37.5)	4 (28.6)	7 (36.8)	3 (14.3)	
Digestive	4 (12.9)	1 (7.1)	4 (11.8)	3 (11.5)		2 (12.5)	1 (7.1)	1 (5.3)	4 (19.0)	
Respiratory	2 (6.5)	1 (7.1)	3 (8.8)	4 (15.4)		0 (0.0)	1 (7.1)	3 (15.8)	4 (19.0)	
Blood/myeloproliferative syn.	1 (3.2)	0 (0.0)	6 (17.6)	2 (7.7)		1 (6.3)	0 (0.0)	0 (0.0)	4 (19.0)	
Renal/urologic	3 (9.7)	0 (0.0)	2 (5.9)	2 (7.7)		2 (12.5)	1 (7.1)	2 (10.5)	1 (4.8)	
Others^c^	2 (6.4)	1 (7.1)	3 (8.7)	1 (3.8)		1 (6.3)	1 (7.1)	2 (10.5)	0 (0.0)	
Functional status score at admission^d^ [median (IQR)]	50 (40.0)	72.5 (32.5)	57.5 (41.3)	72.5 (46.3)	0.250	67.5 (42.5)	55.0 (36.3)	60.0 (40.0)	70.0 (40.0)	0.922
FAC score at admission^§^ [median (IQR)]	3 (2.0)	3.5 (3.0)	3.0 (2.0)	3.0 (2.0)	0.664	2.5 (3.0)	4.0 (1.0)	2.0 (2.0)	3.0 (1.0)	0.137
Independent ambulation at admission^e§^ [n(%)]	14 (46.7)	7 (50.0)	9 (27.3)	12 (46.2)	0.292	6 (37.5)	8 (57.1)	7 (36.8)	9 (42.9)	0.652
SPPB score at admission^§^ [median (IQR)]	3.0 (5.0)	2.0 (4.0)	3.0 (3.8)	3.0 (5.5)	0.290	4.5 (7.0)	2.5 (3.3)	3.0 (5.5)	5.0 (5.5)	0.417
Length of hospitalization^§^ [median (IQR)], d	5.0 (4.0)	4.0 (4.3)	5.0 (3.3)	5.5 (9)	0.108	4.5 (3.3)	5.0 (5.0)	5.0 (4.0)	5.0 (7.0)	0.786
MedDiet adherence score^§^ [mean (SD)]	6.5 (1.4)^a^	9.8 (1.0)^b^	6.7 (1.4)^a^	9.9 (1.0)^b^	<0.001	7.9 (2.2)	7.5 (2.4)	8.7 (2.0)	8.2 (2.0)	0.420
UTP levels at admission^§^ [mean (SD)], mg GAEs/L	10.8 (2.6)	10.7 (1.9)	10.7 (2.7)	10.7 (2.2)	0.999	8.6 (1.3)^a^	12.8 (1.3)^b^	8.5 (2.3)^a^	12.5 (0.8)^b^	<0.001

Note. BMI = body mass index; FAC = functional ambulatory classification; GAEs = gallic acid equivalents; IQR = interquartile range; MedDiet = Mediterranean diet; SD = standard deviation; SPPB = Short Physical Performance Battery; UTP = urinary total polyphenols. a. Frailty was defined as having ≥ 3 of 5 Fried criteria (17). b. Malnutrition was defined as having ≤ 7 of 14 Mini Nutritional Assessment-Short Form criteria (16). c. The “Others” category includes central nervous system, musculoskeletal, endocrine and neoplasia admission diagnosis. d. Functional status was assessed with the Barthel Index of independence in ADLs, which uses a total 0 to 100 score and includes the following 10 basic ADLs: feeding, transferring, walking on level surfaces, stair climbing, bowel and bladder control, toileting, bathing, grooming and dressing (15). e. Independent ambulation was considered in the event of an FAC = 4 (19). f. A low level of MedDiet adherence consisted in a MEDAS score < 9 and a high level of MedDiet adherence consisted in a MEDAS score ≥ 9 at admission. Low levels of UTP consisted in ≤ 11.1 mg GAEs/L and high levels of UTP consisted in > 11.2 mg GAEs/L at admission; § Variables containing missing values. The number of missing values in the different groups [low adherence + control, high adherence + control, low adherence + intervention, high adherence + intervention] [low UTP levels + control, high UTP levels + control, low UTP levels + intervention, high UTP levels + intervention] for the following covariables are: BMI at admission [6, 0, 3, 0] [2, 2, 0, 0]; Charlson comorbidity index [2, 0, 0, 0] [0, 0, 0, 0]; Frailty phenotype [10, 4, 6, 2] [5, 4, 2, 0]; Urinary incontinence [1, 0, 0, 0] [0, 0, 0, 0]; Depression [1, 0, 0, 0] [0, 0, 0, 0]; Falls [1, 0, 0, 0] [0, 0, 0, 0]; Chronic pain [1, 0, 0, 0] [0, 0, 0, 0]; Malnutrition [6, 0, 7, 2] [2, 2, 2, 1]; Dementia [1, 0, 0, 0] [0, 0, 0, 0]; Polypharmacy [1, 0, 0, 0] [0, 0, 0, 0]; FAC score at admission [1, 0, 1, 0] [0, 0, 0, 0]; Independent ambulation at admission [1, 0, 1, 0] [0, 0, 0, 0]; SPPB score at admission [3, 3, 2, 1] [2, 0, 1, 0]; Length of hospitalization [2, 0, 0, 0] [0, 0, 0, 0]; MedDiet adherence score [0, 0, 0, 0] [1, 0, 1, 2]; UTP levels at admission [12, 4, 17, 6] [0, 0, 0, 0]. Values without the same superscript differ (p < 0.05; Bonferroni post hoc test).

Participants in the intervention group presented a significantly better BI at discharge [adjusted mean (95% CI) = 75.7 (71.1–80.3) points] compared to admission [adjusted mean (95% CI) = 67.2 (62.8–71.5) points; p = 0.002] as well as to the control group [adjusted mean (95% CI) = 65.7 (59.4–71.9) points; p = 0.014] (Supplementary Table 2). No significant differences in functional status were noted in the control group during hospitalization (p > 0.05; Supplementary Table 2). The adjusted mean of change (95% CI) of functional status in the control group was 1.2 (−4.5–6.9) points and 9.3 (5.2–13.5) points in the intervention group (p = 0.014; Supplementary Table 2).

Differences in functional status during hospitalization (i.e. BI at discharge vs. BI at admission) between low and high levels of MedDiet adherence and UTP at admission are reported in Figure 1. Participants with low MedDiet adherence in the intervention group significantly increased their BI during hospitalization (Figure 1 and Supplementary Table 3). In addition, in the adjusted model, the functional status at discharge of these participants was significantly higher than that of the control group (p < 0.05). However, we did not observe significant increases in the control group or among those subjects with a high MedDiet adherence (all p > 0.05) (Figure 1 and Supplementary Table 3).

In the same vein, participants with low UTP levels at admission in the intervention group significantly increased their BI during hospitalization (Figure 1 and Supplementary Table 3). No differences in functional status during hospitalization were found among participants from either control group or with high levels of UTP at admission (all p > 0.05) (Figure 1 and Supplementary Table 3).

In addition, in the intervention group, we observed higher changes in BI in the low level of MedDiet adherence [mean (95% CI) of 12.3 (6.0–18.6)] and UTP groups [mean (95% CI) of 13.2 (4.6–21.8)] than in the high-level groups [MedDiet adherence (mean (95% CI)) of 5.8 (−0.8–12.3); UTP (mean (95% CI)) of 9.7 (2.3–17.0)], although they did not reach statistical significance (all p > 0.05) (Supplementary Table 4). It is remarkable that although not significant, changes in the control group were lower than in the intervention group (Supplementary Table 4). All these differences remained significant in sensitivity analyses (Supplementary Tables 2, 4 and 5).

In supplementary analyses, we explored the potential mediation effect of malnutrition on the relationship between MedDiet adherence and UTP levels and the response to the intervention through the Sobel test (24). We did not observe significant mediation pathways between MedDiet adherence or UTP levels and the response to the intervention through malnutrition [t (SE) = −0.174 (0.002), p-value = 0.861 for MedDiet adherence; and t (SE) = 0.522 (0.012), p-value = 0.601 for UTP levels] (25).

## Discussion

To the best of our knowledge, this is the first work with hospitalized older patients aged over 75 to evaluate the relationship between a healthy dietary pattern and functional status following a physical exercise and health education intervention. This secondary analysis shows that, although the intervention with exercise has proven effective and safe overall (4), it has a greater effect in individuals who have low adherence to the MedDiet. In fact, participants who had a low MedDiet adherence significantly increased their functional status by an adjusted mean (95% CI) of 12.3 (6.0–18.6) points in the BI after intervention with exercise, which is considered clinically relevant (26). In contrast, no further differences between BI at admission and at discharge were noted in the control group or among those subjects with a high MedDiet adherence. Multi-domain interventions, where both exercise and nutritional interventions are optimally designed, tend to reveal a stronger effect on frailty, physical functioning, and muscle mass and strength than physical exercise alone (27). However, not all older people may benefit from the same type of exercise interventions in the same way. Valenzuela et al. (2020) reported interindividual variability in the responsiveness to physical exercise intervention in acutely hospitalized older adults. Interestingly, a worse nutritional status at admission was associated with a greater responsiveness to the intervention (28). Furthermore, in a multicentric prospective observational study, researchers observed that improvement in functional status was higher in frail institutionalized older adults with lower vitamin D levels and poorer nutritional status after an oral nutritional supplementation and physical exercise than those with ≥ 20 ng/mL vitamin D plasmatic levels (29). Franzke et al. (2019) observed significant correlations between changes in micronutrient status and changes in performance parameters only among institutionalized older subjects who did not receive protein and vitamin supplementation (30). This is consistent with our results, although to the best of our knowledge, no results have been previously reported regarding baseline dietary patterns rather than nutritional status or nutritional supplementation. Actually, extensive literature supports the MedDiet as a dietary pattern for preventing chronic disease and malnutrition, as well as for maintaining health on aging (31). In this sense, taking into account the obtained results, we can see that those older individuals who were admitted with a low level of MedDiet adherence had a bigger improvement margin when following a simple in-hospital exercise and health education intervention than their peers with higher adherence. This led us to hypothesize that stronger interventions should be carried out among individuals with high MedDiet adherence as not all older patients may require the same exercise intensity to prevent HAD.

In the present study, statistically significant differences were also found in those subjects who were in the intervention group and who showed low levels of UTP at admission. In line with the results obtained for MedDiet adherence, we did not observe differences for the control group or high UTP levels. As far as we are aware, no studies have evaluated levels of UTP in acutely hospitalized older patients, although previous results of the research group obtained in the InCHIANTI cohort have shown that the prevalence of frailty decreased with increasing UTP tertiles in community-dwelling older subjects of a mean age of 73 years, as did the prevalence of disability in more than one ADL and instrumental ADLs (9). Similarly, high UTP concentrations were also associated with a lower risk of physical performance decline and mortality over nine years of follow-up (11, 32). In fact, numerous studies have suggested protective associations between polyphenol intake and some age-related pathologies (33). These protective effects may be attributable to their hermetic functions and their stimulus towards adaptive cellular responses, which, in turn, delay cellular aging.

Some limitations should be considered. This study is a single-center study performed in a frail population (i.e. the proportion of participants who were fully independent in all of the 10 ADLs at baseline was only 17%) and whose mean age (SD) was 87 (5) years, which could restrict the extrapolation of our findings to younger or more functional patients. However, while limited by a small sample size, the main strength of our study is precisely the unusual inclusion of acutely hospitalized older participants aged 75 to 98 with a high proportion of the frailty phenotype (~50%). Another limitation of our study is that assessors, care providers, and participants were unblinded to the intervention with exercise. In addition, the present study population was aged 75 and older and therefore may be less reliable in recalling food intake than younger subjects, although MEDAS is a country-specific questionnaire and has been previously validated for older subjects (13). To account for that, we used UTP as an objective biomarker of polyphenol-rich food intake and thus a proxy biomarker of MedDiet (9). Furthermore, participants with severe dementia at admission were excluded. The cross-sectional design nested in a randomized clinical trial did not allow us to assess causality between UTP or MedDiet adherence and functional status.

A shortcoming of this current investigation is the potential for type II statistical error. The sample size for the AGECAR-PLUS (n = 252; final enrollment n = 260) was selected to permit the detection of a recovery rate up to 60% with a statistical power of 80% (α = 0.05). For the present secondary analysis, the estimated effect size for MedDiet assessed through the MEDAS questionnaire (n = 105) revealed a statistical power of 60%. The estimated effect size for MedDiet assessed through the UTP levels (n = 70) revealed a statistical power of 55%. Given the exploratory nature of this analysis and moderated statistical power, outcomes need to be interpreted cautiously. The impact of MedDiet on the response to the exercise program was a secondary outcome and the AGECAR-PLUS trial was not powered for this outcome. As a result, our findings offer a hypothesis-generator and provide a basis for further research rather than firm evidence. Hence, further studies that include an appropriate sample size should be designed focusing on MedDiet interventions in an ACE context.

Finally, some outcome-influential variables, such as nutritional supplementation, emotional status, lifestyle, and physical activity levels prior to hospitalization, were not directly assessed and therefore our analyses were not controlled for these potential confounders which might have conceivably biased the study outcomes.

## Conclusion

In conclusion, this study showed that hospitalized older patients over age 75 (mean age 87, and range 75–98) with low MedDiet adherence were likely to benefit more from a physical exercise and health education program than those with high values. These results suggest that adherence to the MedDiet could represent a potential indicator of those older patients aged over 75 with a putative better response to exercise interventions. Therefore, the inclusion of MedDiet adherence or UTP levels in the variables assessed at admission of older patients could allow more accurate and personalized strategies to be designed with a view to preventing HAD.

## Supplementary Material

Supplementary material, approximately 236 KB.

*Author contributions:* J.M.-C., J.A.S.-R., C.A.-L. and M.U.-S. conceptualized and designed the research; J.M.-C., A.T.-R., J.A.S.-R. and M.U.-S. carried out the research, analyzed data and performed statistical analysis; A.T.-R. and M.U.-S. wrote the original draft; all the authors critically reviewed and edited this manuscript. All the authors read and approved the final manuscript.

*Funding:* This work was supported by the EU Joint Programming Initiative (JPI) “A Healthy Diet for a Healthy Life - HDHL” ERA-Net Cofund on HDHL-INTIMIC (METADIS) by the Instituto de Salud Carlos III thorough Acción Estratégica en Salud 2020 and within the JPI-HDHL framework Project (AC19/00111); the Instituto de Salud Carlos III and co-funded by the European Regional Development Fund “A way to make Europe” (PI17/02021); the Centro de Investigación Biomédica en Red Fragilidad y Envejecimiento Saludable (CIBERFES), co-funded by the FEDER Program from the EU; the Ministry of Economy and Competitiveness (PID2020-114921RB-C21); the INSA-UB institute recognized as a Maria de Maeztu Unit of Excellence (CEX2021-001234-M) funded by MICINN/AEI/FEDER, EU; and the Generalitat de Catalunya Agency AGAUR (2021SGR00687). AT-R is grateful for the FPU 2019 contract from the Ministry of Science, Innovation and Universities (FPU19/06044), CA-L for the ICREA Academia award 2018 from the Generalitat de Catalunya, and MU-S for the I3 Program from the Ministry of Science and Innovation (IED2019-001061-I). Open Access funding provided thanks to the CRUE-CSIC agreement with Springer Nature.

*Ethics declarations:* Conflict of Interest: All authors have completed the ICMJE uniform disclosure form and declare that the research was conducted in the absence of any commercial or financial relationships that could be construed as a potential conflict of interest.

*Ethical standards:* The Institutional Review Board approved the protocol (Hospital Universitario Gregorio Marañón, Madrid, Spain), and all participants provided written informed consent. When it was not possible to obtain the informed consent directly from a patient for medical reasons (e.g. impaired cognitive function), proxy consent was obtained from their relatives. All procedures were performed in accordance with the ethical standards from the Declaration of Helsinki and its later amendments.
