# Supplementary material for: Adherence to Mediterranean Diet and Response to an Exercise Program to Prevent Hospitalization-Associated Disability in Older Adults: A Secondary Analysis from a Randomized Controlled Trial
Source: J Nutr Health Aging. 2024 Jan 4;27(7):500–6. doi: 10.1007/s12603-023-1929-6 (PMC12929977; doi:10.1007/s12603-023-1929-6)
Supplement: Supplementary file 1 — Supplementary material, approximately 236 KB. [file mmc1.docx]

SUPPLEMENTARY MATERIAL

**Supplementary Figure 1.** CONSORT 2010 Flow Diagram of This Study. ACE = acute care for older patients; BI = Barthel Index; MEDAS = Mediterranean Diet Adherence Score.

**Supplementary Table 1.** Main Characteristics of the Study Participants by Intervention Group and MedDiet Adherence (Complete-Case Analysis).

**Supplementary Table 2.** Functional Status at Admission and at Discharge of Participants According to Intervention Group.

**Supplementary Table 3.** Functional Status at Admission and at Discharge of Participants in Both Control and Intervention Groups According to MedDiet Adherence and UTP Levels (mg GAEs/L) at Admission (Intention-to-Treat Analysis).

**Supplementary Table 4.** Changes in Functional Status During Hospitalization of Participants in Both Control and Intervention Groups According to MedDiet Adherence and UTP Levels (mg GAEs/L) at Admission.

**Supplementary Table 5.** Functional Status at Admission and at Discharge of Participants in Both Control and Intervention Groups According to MedDiet Adherence and UTP Levels (mg GAEs/L) at Admission (Complete-Case Analysis).

**Supplementary Figure 1.** CONSORT 2010 Flow Diagram of This Study. ACE = acute care for older patients; BI = Barthel Index; MEDAS = Mediterranean Diet Adherence Score.


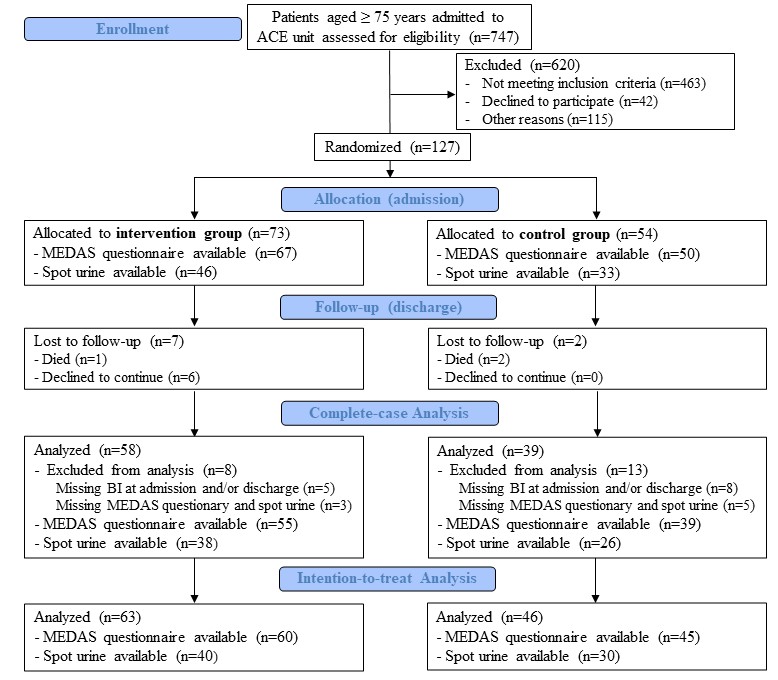


**Supplementary Table 1.** Main Characteristics of the Study Participants by Intervention Group and Mediterranean Diet Adherence (Complete-Case Analysis).

| Variables | | Control | | Intervention | | *p* |  | Control | | Intervention | | *p* |
| --- | --- | --- | --- | --- | --- | --- | --- | --- | --- | --- | --- | --- |
|  |  | Low adherence^f^ (n=26) | High adherence^f^ (n=13) | Low adherence^f^ (n=31) | High adherence^f^ (n=24) |  |  | Low UTP levels^f^ (n=13) | High UTP levels^f^ (n=13) | Low UTP levels^f^ (n=19) | High UTP levels^f^ (n=19) |  |
| Age [mean (SD)], y. | | 88.4 (4.9) | 86.1 (4.2) | 87.3 (4.2) | 87.4 (4.9) | 0.514 |  | 87.4 (5.0) | 86.3 (5.4) | 87.2 (4.6) | 87.5 (4.4) | 0.908 |
| Female [n (%)] | | 13 (50) | 6 (46.2) | 15 (48.4) | 9 (37.5) | 0.817 |  | 6 (46.2) | 7 (53.8) | 7 (36.8) | 8 (42.1) | 0.811 |
| BMI at admission^§^ [mean (SD)], kg/m^2^ | | 20.7 (3.5)^ab^ | 21.4 (4.2)^ab^ | 23.0 (3.8)^a^ | 20.4 (2.4)^b^ | 0.031 |  | 20.0 (2.7) | 23.4 (4.6) | 21.6 (2.6) | 20.6 (4.0) | 0.096 |
| Living at home [n (%)] | | 23 (88.5) | 13 (100) | 29 (93.5) | 23 (95.8) | 0.635 |  | 13 (100) | 12 (92.3) | 17 (89.5) | 18 (94.7) | 0.904 |
| Charlson comorbidity index^§^ [mean (SD)] | | 2.7 (1.8) | 2.3 (1.8) | 3.2 (1.9) | 3.5 (2.7) | 0.290 |  | 2.2 (2.3) | 2.5 (1.4) | 3.0 (2.0) | 3.5 (2.4) | 0.368 |
| *Geriatric syndromes* | |  |  |  |  |  |  |  |  |  |  |  |
|  | Frailty phenotype^a§^ [n (%)] | 14 (73.7) | 8 (80.0) | 14 (53.8) | 14 (63.6) | 0.383 |  | 8 (80.0) | 9 (90.0) | 10 (58.8) | 13 (68.4) | 0.338 |
|  | Urinary incontinence^§^ [n (%)] | 14 (56.0) | 5 (38.5) | 15 (48.4) | 12 (50.0) | 0.783 |  | 7 (53.8) | 6 (46.2) | 12 (63.2) | 10 (52.6) | 0.809 |
|  | Depression^§^ [n (%)] | 10 (40.0) | 6 (46.2) | 12 (38.7) | 8 (33.3) | 0.893 |  | 6 (46.2) | 7 (53.8) | 5 (26.3) | 9 (47.4) | 0.395 |
|  | Falls^§^ [n (%)] | 11 (44.0) | 3 (23.1) | 9 (29.0) | 8 (33.3) | 0.541 |  | 6 (46.2) | 4 (30.8) | 7 (36.8) | 5 (26.3) | 0.693 |
|  | Chronic pain^§^ [n (%)] | 9 (36.0) | 2 (15.4) | 9 (29.0) | 10 (41.7) | 0.396 |  | 5 (38.5) | 3 (23.1) | 9 (47.4) | 6 (31.6) | 0.525 |
|  | Malnutrition^b§^ [n (%)] | 4 (19.0) | 4 (30.8) | 2 (8.0) | 7 (31.8) | 0.074 |  | 3 (25.0) | 2 (18.2) | 4 (23.5) | 5 (27.8) | 0.418 |
|  | Dementia^§^ [n (%)] | 4 (16.0) | 1 (7.7) | 2 (6.5) | 4 (16.7) | 0.642 |  | 1 (7.7) | 1 (7.7) | 3 (15.8) | 3 (15.8) | 0.808 |
| Polypharmacy^§^ (≥7) [n (%)] | | 16 (64.0) | 11 (84.6) | 26 (83.9) | 19 (79.2) | 0.291 |  | 10 (76.9) | 9 (69.2) | 16 (84.2) | 16 (84.2) | 0.720 |
| Main admission diagnosis [n (%)] | |  |  |  |  | 0.907 |  |  |  |  |  | 0.443 |
|  | *Infectious* | 7 (26.9) | 5 (38.5) | 8 (25.8) | 7 (29.2) |  |  | 3 (23.1) | 5 (38.5) | 4 (21.1) | 4 (21.1) |  |
|  | *Circulatory* | 7 (26.9) | 5 (38.5) | 6 (19.4) | 7 (29.2) |  |  | 5 (38.5) | 4 (30.8) | 7 (36.8) | 3 (15.8) |  |
|  | *Digestive* | 4 (15.4) | 1 (7.7) | 4 (12.9) | 3 (12.5) |  |  | 2 (15.4) | 1 (7.7) | 1 (5.3) | 4 (21.1) |  |
|  | *Respiratory* | 2 (7.7) | 1 (7.7) | 3 (9.7) | 3 (12.5) |  |  | 0 (0.0) | 1 (7.7) | 3 (15.8) | 3 (15.8) |  |
|  | *Blood/myeloproliferative syn.* | 1 (3.8) | 0 (0.0) | 5 (16.1) | 1 (4.2) |  |  | 1 (7.7) | 0 (0.0) | 0 (0.0) | 4 (21.1) |  |
|  | *Renal/urologic* | 3 (11.5) | 0 (0.0) | 2 (6.5) | 2 (8.3) |  |  | 1 (7.7) | 1 (7.7) | 2 (10.5) | 1 (5.3) |  |
|  | *Others^c^* | 2 (7.6) | 1 (7.7) | 3 (9.6) | 1 (4.2) |  |  | 1 (7.7) | 1 (7.7) | 2 (10.6) | 0 (0.0) |  |
| Functional status score at admission^d§^ [median (IQR)] | | 47.5 (45.0) | 75.0 (30.0) | 55 (40.0) | 80.0 (48.8) | 0.191 |  | 75.0 (45.0) | 55.0 (37.5) | 60.0 (40.0) | 70.0 (45.0) | 0.943 |
| FAC score at admission^§^ [median (IQR)] | | 3.0 (2.0) | 4.0 (2.5) | 3.0 (1.3) | 3.0 (2) | 0.584 |  | 3.0 (3.0) | 4.0 (1.0) | 2.0 (2.0) | 3.0 (1.0) | 0.296 |
| Independent ambulation at admission^e§^ [n (%)] | | 11 (42.3) | 7 (53.8) | 7 (23.3) | 11 (45.8) | 0.179 |  | 5 (38.5) | 7 (53.8) | 7 (36.8) | 7 (36.8) | 0.755 |
| SPPB score at admission^§^ [median (IQR)] | | 2.0 (6.0) | 2.0 (4.0) | 3.0 (3.5) | 3.5 (5.8) | 0.250 |  | 3.0 (6.5) | 2.0 (3.0) | 3.0 (5.5) | 5.0 (6.0) | 0.415 |
| Length of hospitalization^§^ [median (IQR)], d. | | 5.0 (5.0) | 5.0 (4.5) | 5.0 (3.0) | 5.5 (7.8) | 0.282 |  | 5.0 (3.5) | 5.0 (5.0) | 5.0 (4.0) | 5.0 (4.0) | 0.921 |
| MedDiet adherence score^§^ [mean (SD)] | | 6.5 (1.5)^a^ | 9.7 (0.9)^b^ | 6.7 (1.5)^a^ | 10.0 (1.0)^b^ | <0.001 |  | 7.7 (2.1) | 7.7 (2.4) | 8.7 (2.0) | 8.2 (2.2) | 0.490 |
| UTP levels at admission^§^ [mean (SD)], mg GAEs/L | | 10.9 (2.5) | 10.8 (2.0) | 10.7 (2.8) | 10.6 (2.1) | 0.989 |  | 8.9 (1.0)^a^ | 12.8 (1.4)^b^ | 8.5 (2.3)^a^ | 12.5 (0.8)^b^ | <0.001 |

*Note*. BMI = body mass index; FAC = functional ambulatory classification; GAEs = gallic acid equivalents; IQR = interquartile range; MedDiet = Mediterranean diet; SD = standard deviation; SPPB = Short Physical Performance Battery; UTP = urinary total polyphenols.

^a^ Frailty was defined as having ≥ 3 of 5 Fried criteria ^1^. ^b^ Malnutrition was defined as having ≤ 7 of 14 Mini Nutritional Assessment–Short Form criteria ^2^. ^c^ The “Others” category includes central nervous system, musculoskeletal, endocrine and neoplasia admission diagnosis. ^d^ Functional status was assessed with the Barthel Index of independence in ADLs, which uses a total 0 to 100 score and includes the following 10 basic ADLs: feeding, transferring, walking on level surfaces, stair climbing, bowel and bladder control, toileting, bathing, grooming and dressing ^3^. ^e^ Independent ambulation was considered in the event of an FAC = 4 ^4^. ^f^ A low level of MedDiet adherence consisted in a MEDAS score < 9 and a high level of MedDiet adherence consisted in a MEDAS score ≥ 9 at admission. Low levels of UTP consisted in ≤ 11.1 mg GAEs/L and high levels of UTP consisted in > 11.2 mg GAEs/L at admission.

^§^ Variables containing missing values. The number of missing values in the different groups [low adherence + control, high adherence + control, low adherence + intervention, high adherence + intervention] [low UTP levels + control, high UTP levels + control, low UTP levels + intervention, high UTP levels + intervention] for the following covariables are: BMI at admission [5, 0, 3, 0] [1, 2, 0, 0]; Charlson comorbidity index [1, 0, 0, 0] [0, 0, 0, 0]; Frailty phenotype [7, 3, 5, 2] [3, 3, 2, 0]; Urinary incontinence [1, 0, 0, 0] [0, 0, 0, 0]; Depression [1, 0, 0, 0] [0, 0, 0, 0]; Falls [1, 0, 0, 0] [0, 0, 0, 0]; Chronic pain [1, 0, 0, 0] [0, 0, 0, 0]; Malnutrition [5, 0, 6, 2] [1, 2, 2, 1]; Dementia [1, 0, 0, 0] [0, 0, 0, 0]; Polypharmacy [1, 0, 0, 0] [0, 0, 0, 0]; FAC score at admission [0, 0, 1, 0] [0, 0, 0, 0]; Independent ambulation at admission [0, 0, 1, 0] [0, 0, 0, 0]; SPPB score at admission [3, 2, 2, 0] [1, 0, 1, 0]; Length of hospitalization [1, 0, 0, 0] [0, 0, 0, 0]; MedDiet adherence score [0, 0, 0, 0] [0, 0, 1, 2]; UTP levels at admission [9, 4, 15, 5] [0, 0, 0, 0].

Values without the same superscript differ (*p* < 0.05; Bonferroni post hoc test).

**Supplementary Table 2.** Functional Status at Admission and at Discharge of Participants According to Intervention Group.

|  |  | Barthel Index (points)^a^ | | | | | | | |
| --- | --- | --- | --- | --- | --- | --- | --- | --- | --- |
|  |  | intention-to-treat analysis | | | |  | complete-case analysis | | |
|  |  | Control | | Intervention | |  | Control |  | Intervention |
|  |  | N | mean (95% CI) | N | mean (95% CI) | N | mean (95% CI) | N | mean (95% CI) |
| *Unadjusted model* | | |  |  |  |  |  |  |  |
|  | Admission | 46 | 62.2 (55.0–69.3) | 63 | 64.0 (58.0–70.1) | 39 | 62.9 (55.0–70.9) | 58 | 64.3 (57.8–70.8) |
|  | Discharge | 46 | 64.8 (58.3–71.3) | 63 | 73.5 (67.9–79.1)^**¥^ | 39 | 66.0 (58.9–73.1) | 58 | 74.6 (68.7–80.4)^**¥^ |
|  | Change | 46 | 2.2 (-2.4–6.8) | 63 | 9.7 (5.8–13.7)^¥^ | 39 | 2.8 (-2.4–8.0) | 58 | 10.5 (6.2–14.7^)¥^ |
| *Adjusted model* | | |  |  |  |  |  |  |  |
|  | Admission | 31 | 63.1 (57.2–69.0) | 55 | 67.2 (62.8–71.5) | 29 | 62.1 (55.9–68.3) | 51 | 67.4 (62.8–72.0) |
|  | Discharge | 31 | 65.7 (59.4–71.9) | 55 | 75.7 (71.1–80.3)^*¥^ | 29 | 65.1 (58.6–71.7) | 51 | 76.4 (71.6–81.2)^*¥^ |
|  | Change | 31 | 1.2 (-4.5–6.9) | 55 | 9.3 (5.2–13.5) ^¥^ | 29 | 1.2 (-4.9–7.2) | 51 | 10.1 (5.7–14.6) ^¥^ |

*Note*. CI = confidence interval. Adjusted models are adjusted for sex, age, BMI at admission, place of living, independent ambulation, Charlson Comorbidity Index, polypharmacy (≥ 7), cause of hospitalization (admission diagnosis) and frailty phenotype. In all models of changes in functional status, we adjusted for functional status at admission.

^a^ Functional status was assessed with the Barthel Index of independence in ADLs, which uses a total 0 to 100 score and includes the following 10 basic ADLs: feeding, transferring, walking on level surfaces, stair climbing, bowel and bladder control, toileting, bathing, grooming and dressing ^3^.

* Different from admission, ^*^ *p* < 0.05, ^**^ *p* ≤ 0.001; ¥ Different from control group, ^¥^ *p* < 0.05 (ANOVA for repeated measures with Bonferroni post hoc test).

**Supplementary Table 3.** Functional Status at Admission and at Discharge of Participants in Both Control and Intervention Groups According to MedDiet Adherence and UTP Levels (mg GAEs/L) at Admission (Intention-to-Treat Analysis).

|  | | | Barthel Index (points)^b^ | | | | |
| --- | --- | --- | --- | --- | --- | --- | --- |
|  |  |  | Time | Control | | Intervention | |
|  |  |  |  | N | mean (95% CI) | N | mean (95% CI) |
| MEDAS score at admission | | |  |  |  |  |  |
| *Unadjusted model* | | |  |  |  |  |  |
|  | Low MedDiet adherence^a^ | | Admission | 31 | 59.2 (50.5–67.9) | 34 | 59.4 (51.1–67.8) |
|  |  |  | Discharge | 31 | 63.5 (55.5–71.6) | 34 | 72.2 (64.5–79.9)^**^ |
|  | High MedDiet adherence^a^ | | Admission | 14 | 67.9 (54.8–80.9) | 26 | 69.2 (59.7–78.8) |
|  |  |  | Discharge | 14 | 66.8 (54.8–78.8) | 26 | 74.2 (65.4–83.0) |
| *Adjusted model* | |  |  |  |  |  |  |
|  | Low MedDiet adherence^a^ | | Admission | 21 | 60.5 (53.2–67.7) | 28 | 65.5 (58.8–72.1) |
|  |  |  | Discharge | 21 | 62.9 (55.2–70.5) | 28 | 77.8 (70.8–84.8)^*¥^ |
|  | High MedDiet adherence^a^ | | Admission | 10 | 68.3 (57.7–78.8) | 24 | 68.7 (61.9–75.6) |
|  |  |  | Discharge | 10 | 70.9 (59.8–82.0) | 24 | 72.8 (65.6–80.0) |
|  |  |  |  |  |  |  |  |
| UTP levels at admission | | |  |  |  |  |  |
| *Unadjusted model* | | |  |  |  |  |  |
|  | Low UTP levels^a^ | | Admission | 16 | 60.6 (48.5–72.8) | 19 | 62.4 (51.2–73.5) |
|  |  |  | Discharge | 16 | 67.8 (57.0–78.6) | 19 | 76.8 (67–86.7)^*^ |
|  | High UTP levels^a^ | | Admission | 14 | 60.7 (47.7–73.7) | 21 | 65.7 (55.1–76.3) |
|  |  |  | Discharge | 14 | 62.1 (50.6–73.7) | 21 | 73.8 (64.4–83.2) |
| *Adjusted model* | |  |  |  |  |  |  |
|  | Low UTP levels^a^ | | Admission | 10 | 59.8 (46.5–73.1) | 16 | 66.0 (55.9–76.2) |
|  |  |  | Discharge | 10 | 64.5 (51.8–77.3) | 16 | 78.0 (68.3–87.7)^*^ |
|  | High UTP levels^a^ | | Admission | 10 | 58.2 (44.7–71.7) | 21 | 66.3 (57.7–75.0) |
|  |  |  | Discharge | 10 | 66.5 (53.6–79.5) | 21 | 74.6 (66.3–82.9) |

*Note*. CI = confidence interval; MedDiet = Mediterranean diet; UTP = urinary total polyphenols.

Adjusted models are adjusted for sex, age, BMI at admission, place of living, independent ambulation, Charlson Comorbidity Index, polypharmacy (≥ 7), cause of hospitalization (admission diagnosis) and frailty phenotype. In the UTP levels model, we further adjusted for creatinine clearance at admission and chronic kidney disease.

^a^ A low level of MedDiet adherence consisted in a MEDAS score < 9 and a high level of MedDiet adherence consisted in a MEDAS score ≥ 9 at admission. Low levels of UTP consisted in ≤ 11.1 mg GAEs/L and high levels of UTP consisted in > 11.2 mg GAEs/L at admission. ^b^ Functional status was assessed with the Barthel Index of independence in ADLs, which uses a total 0 to 100 score and includes the following 10 basic ADLs: feeding, transferring, walking on level surfaces, stair climbing, bowel and bladder control, toileting, bathing, grooming and dressing ^3^.

* Different from admission, ^*^ *p* < 0.05, ^**^ *p* ≤ 0.001; ¥ Different from control group, ^¥^ *p* < 0.05 (ANOVA for repeated measures with Bonferroni post hoc test).

**Supplementary Table 4.** Changes in Functional Status During Hospitalization of Participants in Both Control and Intervention Groups According to MedDiet Adherence and UTP Levels (mg GAEs/L) at Admission.

|  |  |  | Changes in Barthel Index (points)^b^ | | | | | | | |
| --- | --- | --- | --- | --- | --- | --- | --- | --- | --- | --- |
|  |  |  | Intention-to-treat analysis | | | | Complete-case analysis | | | |
|  |  |  | Control | | Intervention | | Control | | Intervention | |
|  |  |  | N | mean (95% CI) | N | mean (95% CI) | N | mean (95% CI) | N | mean (95% CI) |
| MEDAS score at admission | | |  |  |  |  |  |  |  |  |
| *Unadjusted model* | | |  |  |  |  |  |  |  |  |
|  | Low MedDiet adherence^a^ | | 31 | 3.1 (-2.7–8.8) | 34 | 11.6 (6.1–17.1) | 26 | 3.7 (-2.8–10.1) | 31 | 12.3 (6.3–18.2) ^¥^ |
|  | High MedDiet adherence^a^ | | 14 | 0.6 (-7.9–9.2) | 26 | 7.2 (0.9–13.5) | 13 | 1.3 (-7.9–10.5) | 24 | 8.0 (1.2–14.8) |
| *Adjusted model* | |  |  |  |  |  |  |  |  |  |
|  | Low MedDiet adherence^a^ | | 21 | -0.2 (-7.2–6.8) | 28 | 12.3 (6.0–18.6) | 19 | -0.3 (-7.9–7.3) | 26 | 13.4 (6.7–20.1) |
|  | High MedDiet adherence^a^ | | 10 | 4.1 (-5.9–14.1) | 24 | 5.8 (-0.8–12.3) | 10 | 3.7 (-6.6–14.0) | 22 | 6.4 (-0.6–13.3) |
|  |  |  |  |  |  |  |  |  |  |  |
| UTP levels at admission | | |  |  |  |  |  |  |  |  |
| *Unadjusted model* | | |  |  |  |  |  |  |  |  |
|  | Low UTP levels^a^ | | 16 | 6.4 (-1.7–14.4) | 19 | 14.4 (7.0–21.7) | 13 | 8.3 (-1.0–17.5) | 19 | 14.4 (6.7–22.0) |
|  | High UTP levels^a^ | | 14 | 0.7 (-7.9–9.3) | 21 | 9.3 (2.3–16.4) | 13 | 0.5 (-8.8–9.7) | 19 | 10.2 (2.5–17.8) |
| *Adjusted model* | |  |  |  |  |  |  |  |  |  |
|  | Low UTP levels^a^ | | 10 | 2.7 (-8.6–14.0) | 16 | 13.2 (4.6–21.8) | 9 | 4.0 (-8.0–16.1) | 16 | 12.9 (4.1–21.6) |
|  | High UTP levels^a^ | | 10 | 5.4 (-6.1–16.9) | 21 | 9.7 (2.3–17.0) | 10 | 5.2 (-6.7–17.2) | 19 | 10.6 (2.6–18.6) |

*Note*. CI = confidence interval; MedDiet = Mediterranean diet; UTP = urinary total polyphenols. All models are adjusted for functional status at admission. Adjusted models are further adjusted for sex, age, BMI at admission, place of living, independent ambulation, Charlson Comorbidity Index, polypharmacy (≥ 7), cause of hospitalization (admission diagnosis) and frailty phenotype. In the UTP levels model, we further adjusted for creatinine clearance at admission and chronic kidney disease.

^a^ A low level of MedDiet adherence consisted in a MEDAS score < 9 and a high level of MedDiet adherence consisted in a MEDAS score ≥ 9 at admission. Low levels of UTP consisted in ≤ 11.1 mg GAEs/L and high levels of UTP consisted in > 11.2 mg GAEs/L at admission. ^b^ Functional status was assessed with the Barthel Index of independence in ADLs, which uses a total 0 to 100 score and includes the following 10 basic ADLs: feeding, transferring, walking on level surfaces, stair climbing, bowel and bladder control, toileting, bathing, grooming and dressing ^3^.

¥ Different from control group, ^¥^ *p* < 0.05 (ANOVA for repeated measures with Bonferroni post hoc test).

**Supplementary Table 5.** Functional Status at Admission and at Discharge of Participants in Both Control and Intervention Groups According to MedDiet Adherence and UTP Levels (mg GAEs/L) at Admission (Complete-Case Analysis).

|  | | | Barthel Index (points)^b^ | | | | |
| --- | --- | --- | --- | --- | --- | --- | --- |
|  |  |  | Time | Control | | Intervention | |
|  |  |  |  | N | mean (95% CI) | N | mean (95% CI) |
| MEDAS score at admission | | |  |  |  |  |  |
| *Unadjusted model* | | |  |  |  |  |  |
|  | Low MedDiet adherence^a^ | | Admission | 26 | 59.4 (49.7–69.1) | 31 | 58.9 (50.0–67.8) |
|  |  |  | Discharge | 26 | 64.6 (55.8–73.5) | 31 | 72.9 (64.8–81.0)^**^ |
|  | High MedDiet adherence^a^ | | Admission | 13 | 70.0 (56.3–83.7) | 24 | 70.4 (60.3–80.5) |
|  |  |  | Discharge | 13 | 68.8 (56.3–81.3) | 24 | 75.8 (66.6–85.0) |
| *Adjusted model* | |  |  |  |  |  |  |
|  | Low MedDiet adherence^a^ | | Admission | 19 | 59.3 (51.5–67.0) | 26 | 65.8 (58.9–72.8) |
|  |  |  | Discharge | 19 | 62.3 (54.1–70.5) | 26 | 78.9 (71.5–86.2)^*¥^ |
|  | High MedDiet adherence^a^ | | Admission | 10 | 66.8 (56.2–77.5) | 22 | 68.9 (61.7–76.0) |
|  |  |  | Discharge | 10 | 69.6 (58.4–80.9) | 22 | 73.2 (65.7–80.7) |
|  |  |  |  |  |  |  |  |
| UTP levels at admission | | |  |  |  |  |  |
| *Unadjusted model* | | |  |  |  |  |  |
|  | Low UTP levels^a^ | | Admission | 13 | 61.2 (47.2–75.1) | 19 | 62.4 (50.8–73.9) |
|  |  |  | Discharge | 13 | 70.0 (57.7–82.3) | 19 | 76.8 (66.7–87.0)^*^ |
|  | High UTP levels^a^ | | Admission | 13 | 60.0 (46.1–73.9) | 19 | 65.5 (54.0–77.1) |
|  |  |  | Discharge | 13 | 61.5 (49.3–73.8) | 19 | 74.5 (64.3–84.6) |
| *Adjusted model* | |  |  |  |  |  |  |
|  | Low UTP levels^a^ | | Admission | 9 | 58.5 (44.6–72.5) | 16 | 65.7 (55.5–75.9) |
|  |  |  | Discharge | 9 | 65.4 (51.9–78.8) | 16 | 77.4 (67.6–87.2)^*^ |
|  | High UTP levels^a^ | | Admission | 10 | 56.3 (42.6–70.0) | 19 | 68.1 (58.9–77.3) |
|  |  |  | Discharge | 10 | 65.5 (52.4–78.7) | 19 | 76.2 (67.4–85.1) |

*Note*. CI = confidence interval; MedDiet = Mediterranean diet; UTP = urinary total polyphenols. Adjusted models are adjusted for sex, age, BMI at admission, place of living, independent ambulation, Charlson Comorbidity Index, polypharmacy (≥ 7), cause of hospitalization (admission diagnosis) and frailty phenotype. In the UTP levels model, we further adjusted for creatinine clearance at admission and chronic kidney disease.

^a^ A low level of MedDiet adherence consisted in a MEDAS score < 9 and a high level of MedDiet adherence consisted in a MEDAS score ≥ 9 at admission. Low levels of UTP consisted in ≤ 11.1 mg GAEs/L and high levels of UTP consisted in > 11.2 mg GAEs/L at admission. ^b^ Functional status was assessed with the Barthel Index of independence in ADLs, which uses a total 0 to 100 score and includes the following 10 basic ADLs: feeding, transferring, walking on level surfaces, stair climbing, bowel and bladder control, toileting, bathing, grooming and dressing ^3^.

* Different from admission, ^*^ *p* < 0.05, ^**^ *p* ≤ 0.001; ¥ Different from control group, ^¥^ *p* < 0.05 (ANOVA for repeated measures with Bonferroni post hoc test).

# Supplementary References

1. Fried LP, Tangen CM, Walston J, et al. Frailty in older adults: Evidence for a phenotype. *Journals of Gerontology - Series A Biological Sciences and Medical Sciences*. 2001;56(3):146-157. doi:10.1093/gerona/56.3.m146

2. Kaiser MJ, Bauer JM, Ramsch C, et al. Validation of the Mini Nutritional Assessment short-form (MNA-SF): a practical tool for identification of nutritional status. *J Nutr Health Aging*. 2009;13(9):782-788.

3. Mahoney FI, Barthel DW. Functional evaluation: the Barthel Index. *Md State Med J*. 1965;14:61-65.

4. Holden MK, Gill KM, Magliozzi MR, Nathan J, Piehl-baker L. Clinical gait assessment in the neurologically impaired. Reliability and meaningfulness. *Phys Ther*. 1984;64(1):35-40. doi:10.1093/ptj/64.1.35
